# Therapeutic Effects of Human Multilineage-Differentiating Stress Enduring (MUSE) Cell Transplantation into Infarct Brain of Mice

**DOI:** 10.1371/journal.pone.0116009

**Published:** 2015-03-06

**Authors:** Tomohiro Yamauchi, Yasumasa Kuroda, Takahiro Morita, Hideo Shichinohe, Kiyohiro Houkin, Mari Dezawa, Satoshi Kuroda

**Affiliations:** 1 Department of Neurosurgery, Hokkaido University Graduate School of Medicine, Sapporo, Japan; 2 Department of Stem Cell Biology and Histology, Graduate School of Medicine, Tohoku University, Sendai, Japan; 3 Department of Neurosurgery, Graduate School of Medicine and Pharmacological Science, University of Toyama, Toyama, Japan; National Institutes of Health, UNITED STATES

## Abstract

**Objective:**

Bone marrow stromal cells (BMSCs) are heterogeneous and their therapeutic effect is pleiotropic. Multilineage-differentiating stress enduring (Muse) cells are recently identified to comprise several percentages of BMSCs, being able to differentiate into triploblastic lineages including neuronal cells and act as tissue repair cells. This study was aimed to clarify how Muse and non-Muse cells in BMSCs contribute to functional recovery after ischemic stroke.

**Methods:**

Human BMSCs were separated into stage specific embryonic antigen-3-positive Muse cells and -negative non-Muse cells. Immunodeficient mice were subjected to permanent middle cerebral artery occlusion and received transplantation of vehicle, Muse, non-Muse or BMSCs (2.5×10^4^ cells) into the ipsilateral striatum 7 days later.

**Results:**

Motor function recovery in BMSC and non-Muse groups became apparent at 21 days after transplantation, but reached the plateau thereafter. In Muse group, functional recovery was not observed for up to 28 days post-transplantation, but became apparent at 35 days post-transplantation. On immunohistochemistry, only Muse cells were integrated into peri-infarct cortex and differentiate into Tuj-1- and NeuN-expressing cells, while negligible number of BMSCs and non-Muse cells remained in the peri-infarct area at 42 days post-transplantation.

**Conclusions:**

These findings strongly suggest that Muse cells and non-Muse cells may contribute differently to tissue regeneration and functional recovery. Muse cells may be more responsible for replacement of the lost neurons through their integration into the peri-infarct cortex and spontaneous differentiation into neuronal marker-positive cells. Non-Muse cells do not remain in the host brain and may exhibit trophic effects rather than cell replacement.

## Introduction

Cell transplantation therapy has been expected to promote functional recovery in various kinds of central nervous system (CNS) disorders including cerebral infarct. The bone marrow stromal cells (BMSCs) may have the enormous therapeutic potential because they can be harvested from the patients themselves and donors without posing ethical or immunological difficulties [1–3]. Based on recent knowledge, allogeneic BMSC transplantation may also be available [4]. More importantly, they are non-tumorigenic and are already applied to the patients with CNS disorders, thus they are highly feasible [5]. The BMSCs are non-hematopoietic cells and are also known as mesenchymal stromal cells [1,2]. For the decades, numerous numbers of studies have indicated that the transplanted BMSCs enhance motor function recovery after the insults in animal models of various neurological disorders, including cerebral infarct [3,6–9]. They also have the potential to ameliorate cognitive dysfunction under certain conditions in diffuse axonal injury and chronic cerebral ischemia models [10,11]. However, there are many variables that may affect the efficacy of BMSC transplantation in the clinical setting. They include donor cell factors (safety, autologous or allogeneic, ex vivo cell expansion), patient factors (age, stroke type), treatment factors (interval since onset, delivery route, cell dose), and validation factors (neurological assessment, imaging) [1]. More importantly, the mechanisms through which the BMSCs promote functional recovery should be clarified. Thus, these functional recoveries may be based on pleiotropic effects of BMSCs, including inflammation modulation and production of neurotrophic factors, as well as replacement of lost neuronal cells by neuronal differentiation of BMSCs. Such multiple properties may result form heterogeneity of BMSCs [12]. Since the geometry of BMSCs is still obscure, however, the cells responsible for neuronal differentiation are not clarified yet. Nevertheless, if the cells that can be integrated into the damaged CNS tissue and spontaneously differentiate into neuronal cells are identified in BMSCs, those would be ideal for regenerative medicine of CNS disorders, and would be expected to improve the efficiency of currently performed BMSC transplantation [1,2].

Recently, multilineage-differentiating stress enduring (Muse) cells are identified in BMSCs [13]. They correspond to several percentages of total BMSCs, and can be efficiently isolated as cells positive for well-known human embryonic stem (ES) cell marker, stage specific embryonic antigen-3 (SSEA-3). Muse cells can self-renew, express a set of genes associated with pluripotency such as Nanog, Oct3/4 and Sox2, and are able to differentiate into endodermal-, ectodermal-, and mesodermal-lineage cells from a single cells. Under cytokine induction, Muse cells differentiate into neuronal maker positive cells with very high ratio of ~90% [14]. Interestingly, they act as tissue repair cells when transplanted *in vivo*; they migrate toward and home into damaged tissues and spontaneously differentiate into cells compatible with the homed-into tissue in fulminant hepatitis, muscle degeneration and skin injury models [13]. Unlike well-known pluripotent stem cells such as ES cells and induced pluripotent stem (iPS) cells, their telomerase activity is low and do not form teratoma in immunodeficient mice testes [14,15]. In contrast, the remainder of BMSCs, non-Muse cells, does not originally express pluripotency genes, nor do they self-renew, differentiate into triploblastic lineages or function as tissue repair cells *in vivo* [14,15]. These results strongly suggest that Muse cells may play a major role in the neural differentiation and thus may directly contribute to tissue regeneration of damaged CNS, although they are only several percentage of total BMSCs. In the past decade, most of transplantation experiment of BMSCs into ischemia model have been conducted by a mixture of heterogeneous BMSCs, and analysis based on a certain subpopulation in BMSCs have not been focused yet.

In this study, therefore, the authors separated human BMSCs into Muse and non-Muse cells, and transplanted each of them into focal cerebral ischemia model to analyze their contribution to tissue regeneration and functional recovery. They also compared the effect exerted by Muse and non-Muse cell transplantation with that of regular BMSC transplantation.

## Materials and Methods

### Cell preparation

Human BMSCs were purchased from Lonza Co. The cells were plated at 5.0×10^5^/75cm^2^ in non-coated flask (EasyFlask 159910; Nunc) in α-MEM (Sigma), 10% fetal bovine serum (FBS; Gibco), and 1% kanamycin (Invitrogen). They were incubated at 37°C and 5%CO_2_. The culture medium was replaced 3 times a week. When the cells were grown to confluence, the cells were lifted by 0.25% trypsin and 0.02% EDTA in PBS. The cells were passed 3 times before cell sorting. To obtain Muse cells, human BMSCs were incubated with rat anti-SSEA-3 IgM antibody (1:50; Millipore, Billerica, MA; detected by fluorescein isothiocyanate-conjugated anti-rat IgM, Jackson Immunoresearch, West Grove, PA) in the FACS antibody diluents and sorted by Special Order Research Products FACSAriaII (Becton Dickinson, Franklin Lakes, NJ) as described previously [13,14]. Cells negative for SSEA-3 were collected as non-Muse cells, and cells unsorted were used as BMSCs.

For preparation of green-fluorescent protein (GFP)-labeled cells, BMSCs were introduced with GFP-lentivirus at the efficiency of ~80% as described previously. GFP-expressing BMSCs (GFP-BMSCs) were selected by the expression of GFP by FACS, and GFP-Muse (GFP (+) / SSEA-3 (+)) and non-Muse (GFP (+) / SSEA-3 (-)) were separated by the expression of SSEA-3 and GFP as described by Kuroda et al. (2010) [13].

### Mice permanent middle cerebral artery occlusion model

All animal experiments were approved by the Animal Study Ethical Committee of Hokkaido University Graduate School of Medicine. Male 6-week-old severe combined immunodeficiency (SCID) mice (n = 24) were purchased from CLEA Japan, Inc. (Tokyo, Japan). Permanent middle cerebral artery (MCA) occlusion was induced as described previously with minor modifications [16,17]. Briefly, anesthesia was induced with 4.0% isoflurane in N_2_O:O_2_ (70:30) and maintained with 2.0% isoflurane in N_2_O:O_2_ (70:30). A 1.0-cm vertical skin incision was made between the right eye and ear, and the temporal muscle was mobilized. Under surgical microscope, a 2.0-mm burr hole was made just on the MCA, using a small dental drill. The dura mater was kept intact, and the right MCA was ligated using 10–0 nylon thread through the dura mater. Core temperature was kept between 36.5 and 37.5°C during and after the procedures. Only animals that circles towards the paretic side were included in this study. Triphenyltetrazolium chloride (TTC; Sigma) staining was performed to quantify infarct volume at 24 hr after the onset of ischemia (n = 4) [18]. Infarct volume was quantified in each animal according to the method described previously [19].

### Cell transplantation

Vehicle, Muse cells, non-Muse cells, or BMSCs (n = 5 in each group) were transplanted into the ipsilateral striatum at 7 days after the onset of permanent MCA occlusion [17,20]. The timing of transplantation was determined according to previous data that the BMSC given 7 days after injury led to significantly larger numbers of surviving cells than immediate treatment and significant improvements of gait [21]. In fact, we have confirmed that the BMSCs significantly promote functional recovery after ischemic stroke when directly injected into the ipsilateral striatum at 7 or 28 days after the onset [22–24]. Briefly, the animals were fixed to a stereotactic apparatus under deep anesthesia with 4.0% isoflurane in N_2_O:O_2_ (70:30), and the cranium was exposed through midline skin incision. A burr hole was made 2 mm right to the bregma, using a small dental drill. A Hamilton syringe was inserted 3 mm into the brain parenchyma from the surface of the dura mater, and 10 μL of cell suspension (2.5×10^4^ cells) or 10 μL of vehicle (PBS) were introduced into the striatum during a period of 5 minutes, using an automatic microinjection pump. Cell dose was determined on the basis of previous data; 1~2 x 10^5^ of BMSCs can significantly promote functional recovery after ischemic stroke in the rats whose brain is ~2.0g. The weight of mouse brain is ~0.4g and therefore we transplanted 2.5×10^4^ cells in this experiment [22,25,26].

### Motor function test

Motor function of the animals was serially assessed before and at 1, 7, 14, 21, 28, 35, 42, and 49 days after the onset of ischemia, using a Rotarod treadmill. This behavioral test was performed in all the Muse cell-, non-Muse cell-, BMSC-, and vehicle-treated mice. The Rotarod was set to the acceleration mode from 4 to 40 rpm for 3 minutes. The maximum time that the animal stayed on the Rotarod was recorded for each performance [27].

### Spatial memory test

Using an eight-arm radial maze test, spatial memory was serially examined before and at 7 and 35 days after the onset of ischemia. The maze consisted of a central platform (24 cm in diameter) with eight arms that extended radially. The mice were allowed to visit each arm to eat eight pellets in food cups located near the end of each arms. Each animal was trained once per day to memorize the apparatus. Their performance in each trial was assessed using two parameters: number of correct choices in the initial eight chosen arms, and number of errors (defined as choosing arm that had already been visited). When the animals made seven or eight correct choices and no more than one error in three successive sessions, they were deemed to have memorized the maze. In other words, the animals had acquired spatial memory of the eight-arm radial maze. [28]

### Histological analysis

At 42 days after transplantation, the animals were deeply anesthetized with 4.0% isoflurane in N_2_O:O_2_ (70:30) and transcardially perfused with 4% paraformaldehyde. The brain was removed, immersed in 4% paraformaldehyde for another 2 days, and 10 μm thick cryosections were made. They were then incubated with block solution, and reacted with primary antibody against human mitochondria (mouse IgG, 1:100, Abcam), GFP (chicken IgG, 1:1000; Abcam), Tuj-1 (mouse IgG, 1:200, Sigma), NeuN (mouse IgG, 1:1000, Chemicon,) and GFAP (mouse IgG, 1:300; Sigma). Samples were further incubated with secondary antibodies either to anti mouse IgG or chicken IgG conjugated with Alexa Fluor 488 or 568 (Invitrogen) and counterstained with DAPI (Invitrogen). Then they were examined using a c1si Nikon confocal microscope system (Nikon, Tokyo, Japan). Using the coronal slice at the level of the striatum (3 slices for each animal), 18 ROIs (800μm x 800μm) were placed in the dorsal neocortex adjacent to the cerebral infarct to count the number of human mitochondria, Tuj-1, NeuN and GFAP-positive cells in each animal.

### Statistical analysis

All data were expressed as mean ± SD. Continuous data were compared by one-factor analysis of variance (ANOVA) followed by Bonferroni’s test among 4 groups. Values of P<0.05 were considered statistically significant. A priori power analysis was employed to determine total sample size, using G*Power Software version 3.1.

## Results

### Infarct volume of permanent MCA occlusion in SCID mice

**Fig 1:**
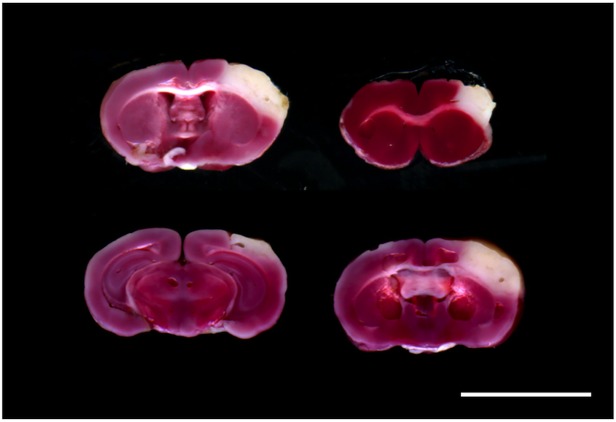
Representative coronal sections of infarct area on triphenyltetrazolium chloride (TTC) staining at 24 hr after permanent middle cerebral artery occlusion. Infarct volume was measured as 22.9 ± 2.9% of the contralateral hemisphere. Scale bar = 5 mm.

All animals could survive after the onset of cerebral ischemia through the experiment. At 24 hr after the onset of ischemia, cerebral infarct was widely distributed in the ipsilateral neocortex. Infarct volume was measured as 22.9 ± 2.9% of the contralateral hemisphere on TTC staining (Fig. 1).

### Effect of cell transplantation on motor function recovery

**Fig 2:**
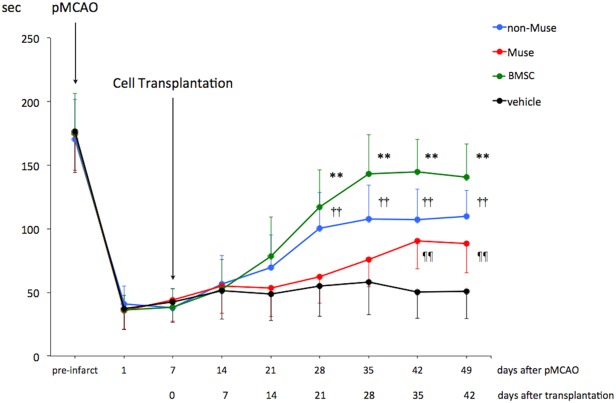
Rotarod treadmill performance. Line graph shows the temporal profile of functional recovery in vehicle-, BMSC-, non-Muse cell-, and Muse cell-treated mice subjected to permanent middle cerebral artery occlusion (pMCAO). **, ††, ¶¶ P<0.01 vs. vehicle-treated mice. Sec indicates seconds.

As shown in Fig. 2, all animals exhibited severe neurological deficit during 7 days after the onset of focal cerebral ischemia. There was no significant difference in motor function among 4 experimental groups. At 7 days after ischemia, cell transplantation was performed. The vehicle group did not show any significant improvement of motor function throughout the experiment. BMSC-transplanted animals started to show functional recovery at 21 days after transplantation, compared with vehicle-transplanted animals (P<0.01). Stereotactic transplantation of non-Muse cells also significantly promoted functional recovery at 21 days after transplantation (P<0.01), but did not show any significant improvement thereafter. Motor function at 28, 35 and 42 days post-transplantation was significantly better in BMSC group than in non-Muse cell group (P<0.05). In Muse cell group, any therapeutic effect was not observed for up to 28 days after transplantation. However, significant improvement became apparent after 35 days post-transplantation, compared with vehicle group (P<0.01), which was later than BMSC and non-Muse cell groups. Motor function at 35 and 42 days post-transplantation was significantly better in BMSC group than in Muse cell group (P<0.05). There were no significant differences in motor function between Muse and non-Muse groups at 35 and 42 days post-transplantation.

### Effect of cell transplantation on spatial memory

**Fig 3:**
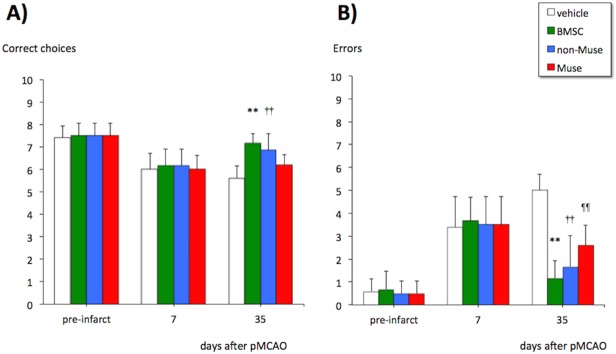
Effects of cell therapy with vehicle, BMSCs, non-Muse cells, and Muse cells on memory impairment at 7 and 35 days after permanent middle cerebral artery occlusion (pMCAO). Correct choices (A) and errors (B) in the eight-arm radial maze task. **, ††, ¶¶ P<0.01 vs. vehicle-treated mice.

The mice subjected to permanent MCA occlusion showed a decrease in the number of correct choices and an increase in the number of errors at 7 days after the onset of ischemia (Fig. 3). Stereotactic transplantation of BMSCs and non-Muse cells, but not of Muse cells, significantly increased the number of correct choices at 4 weeks post-transplantation, compared with the vehicle-transplanted animals. However, stereotactic transplantation of BMSCs, non-Muse cells, and Muse cells significantly decreased the number of errors at the same timing at the same timing (Fig. 3).

### Detection of transplanted cells in the host brain

**Fig 4:**
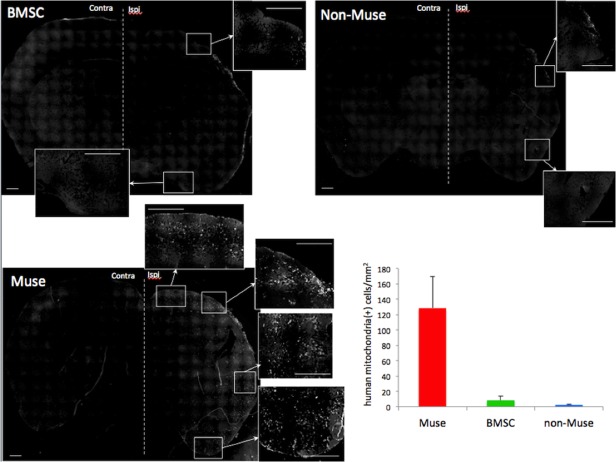
Low-power photomicrographs of fluorescence immunohistochemistry using anti-human mitochondria antibody in BMSC-, non-Muse cell-, and Muse cell-treated mice at 42 days after transplantation. A large number of human mitochondria-positive cells are engrafted in the peri-infarct area in Muse cell group. Graph shows number of human mitochondria-positive cells/mm^2^ in ipsilateral cortex of each group. Scale bars = 500 μm.

Histological analysis was performed at 42 days post-transplantation, *i*.*e*., at 49 day post-ischemia. Transplanted human BMSCs, Muse and non-Muse cells were detected by anti-human mitochondria. BMSC and non-Muse cell groups did not show any efficient integration of the transplanted cells (Fig. 4). Green fluorescent signals were seen in the peri-infarct area; the area that is located adjacent to the lost lesion. However, these were in most cases autofluorescence of phagocytic cells and were not by the immunoreactions to human mitochondria, thus the number of integrated human BMSCs or non-Muse cells was considered negligible (Fig. 4). In sharp contrast, numerous numbers of integrated human cells were widely distributed in the peri-infarct area of the ipsilateral hemisphere in Muse cell group. The number of human mitochondria-positive cells was 128.3 ± 41/mm^2^, which was significantly higher number than that in BMSC- and non-Muse cell groups, 8.5 ± 5.0/mm^2^ and 2.5 ± 0.8/mm^2^, respectively (P<0.01, Fig. 4).

**Fig 5:**
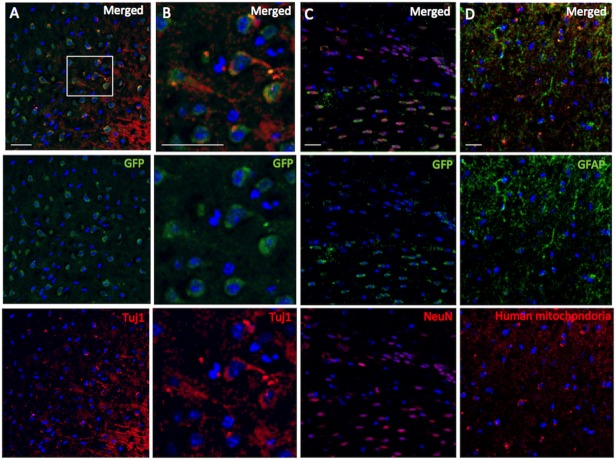
Double immunohistochemistry of GFP-Tuj-1 (A and B), GFP-neuronal nuclear antigen (NeuN; C) and human mitochondria-GFAP (D) in ipsilateral cortex of Muse cell-group (42 days after transplantation). The white square in panel A represents the location of Panel B. Scale bars = 50 μm.

**Fig 6:**
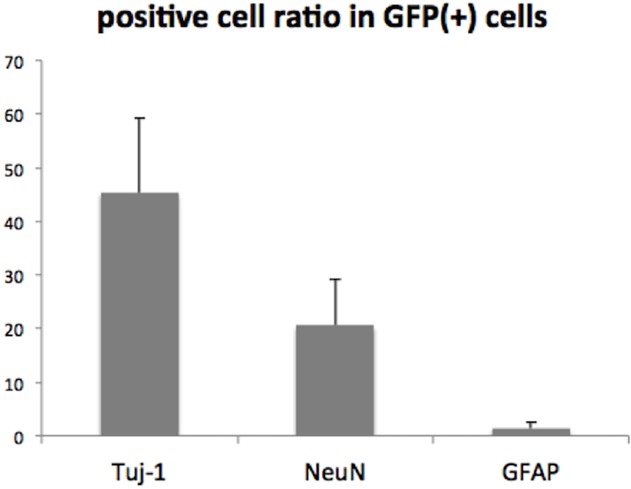
Column graph shows the percentages of Tuj1-, NeuN- and GFAP-positive cells in GFP(+) cells in Muse group (42 days after transplantation).

Fluorescence immunostaining of Muse cell group samples demonstrated that the GFP-positive transplanted human Muse cells distributed in the peri-infarct area expressed neuronal markers, Tuj-1 (45.3 ± 13.9% of total GFP+ cells, Fig. 5A and B, Fig. 6) and NeuN (20.5 ± 8.7% of total GFP+ cells Fig. 5C, Fig. 6). However, only a small number of human mitochondria-positive Muse cells were positive for astrocyte/neural stem cell marker, GFAP (1.4 ± 1.2%; Fig. 5D, Fig. 6), suggesting that majority of integrated Muse cells spontaneously differentiated into neuronal marker-positive cells.

## Discussion

Although further analysis would be necessary, the present data provide important information on the mechanisms through which the engrafted BMSCs promote functional recovery after ischemic stroke. This study clearly demonstrates that at least two subpopulations in BMSCs, Muse and non-Muse cells, may contribute differently to functional recovery when they are directly transplanted into the infarct brain. Non-Muse cells are considered to play a key role in early phase recovery of motor function after transplantation, because the non-Muse cell-transplanted animals started to recover motor function as early as 21 days after transplantation, while their therapeutic effects were limited and did not further promote functional recovery over 42 days. Histological analysis supports the findings. Immunostaining against human mitochondria detects negligible number of non-Muse cells, suggesting that they do not effectively remain or are not integrated into the host brain or even though they are once integrated, they might have disappeared by 42 days after transplantation.

On the other hand, Muse cell-treated animals did not show significant functional recovery during 28 days after transplantation, but started to recover motor function at 35 days post-transplantation, which was later than non-Muse cell-treated animals. On immunohistochemistry, however, a large number of human mitochondria- or GFP-positive cells were shown to engrafted and integrated into the peri-infarct area of ipsilateral hemisphere. There is possibility that some injected Muse cells were phagocytosed, while comparing remaining cell numbers in Muse, BMSC and non-Muse groups, Muse group showed substantial number of remaining cells with significant statistical difference to other two groups. Since cells were directly injected into the striatum by single shot, these integrated Muse cells might have migrated from the striatum into the peri-infarct area. Previous studies have shown that the transplanted cells aggressively migrate towards the lesion through the system of chemokines such as stromal cell-derived factor (SDF)-1 [24]. Majority of integrated Muse cells in the peri-infarct area expressed neuron-specific markers, Tuj-1 and NeuN at 42 days post-transplantation. Nearly 45% of GFP-labeled human Muse cells expressed Tuj-1, and about 20% of them were positive for NeuN, while the Muse cells doubly positive for GFAP was only ~1%, suggesting that Muse cells may preferentially differentiate into neuronal-lineage cells. We have previously shown that Muse cells express nestin, Musashi-1, NeuroD, and MAP-2 after neural induction, which correlates well with present results [14,29]. Longer evaluation of motor function is expected to find further improvement of motor function in Muse cell group.

Because of the sharp contrast between Muse cells and non-Muse cells in functional and histological analysis, it is most likely that these two BMSC subpopulations play biologically different roles in the infarct brain and contribute to post-stroke recovery of motor function and tissue repair by different ways and different timings. Since non-Muse cells did not remain in the host brain, early phase recovery observed in non-Muse cell group may result from the trophic effect exerted by transplanted non-Muse cells rather than cell replacement. In contrast, Muse cells are more responsible for replacement of the lost neuronal cells by integration into the host brain and spontaneous differentiation into neuronal lineage cells.

BMSCs that is consisted of several percentage of Muse cells plus vast majority of non-Muse cells significantly improved motor function by 21 days post-transplantation and yielded better therapeutic effects at 42 days than Muse cells and non-Muse cells. However, the number of integrated BMSCs was very small compared to Muse cells (Fig. 4). At least in short-term evaluation up to 49 days in functional recovery, the BMSCs look as the best to transplant because the mixture of biologically various subpopulations of cells may exert maximal therapeutic effects against ischemic stroke. Nevertheless, longer period observation would be necessary to evaluate therapeutic effects of Muse cells on motor function, because the number of BMSCs integrated into the brain was very small.

Very recent report has provided interesting information on the behaviors of BMSCs engrafted into the infarct brain. Fluorescence in situ hybridization (FISH) studies showed that about half of the engrafted BMSCs express mRNA for brain-derived neurotrophic factor (BDNF) and nerve growth factor (NGF) at 14 days after transplantation into the infarct brain; however, their percentages rapidly decreased thereafter. Instead, the percentage of microtubule-associated protein (MAP) 2–positive BMSCs gradually increased during 28 days after transplantation. These findings strongly support our results suggesting that the BMSCs may exhibit the trophic effect in the early (~2 weeks) stage of cell therapy and the phenotypic change toward neural cells thereafter, when transplanted into the infarct brain [23]. Previous reports have suggested that a certain subpopulation of transplanted BMSCs are integrated in the brain of rodent stroke model [2]. Since Muse cells have the ability to differentiate into neuronal cells both *in vitro* and *in vivo* while non-Muse cells do not show such neuronal differentiations[15], it is plausible small number of neuronal differentiation observed in BMSCs transplantation might be due to Muse cells.

Pathophysiological mechanism of post-stroke cognitive impairment is quite complicated [30]. However, recent studies have shown that the engrafted BMSCs may contribute to improve cognitive function in rodent models of chronic cerebral ischemia, although the underlying mechanisms are still unclear [10]. Indeed, both the BMSCs and non-Muse cells significantly increase the correct choice and decrease the error choice at 28 days after transplantation. Muse cells also decrease the error choice. Likewise the results in Rotarod test, longer evaluation of spatial memory may detect further improvement in the Muse cell-treated animals. It would be quite valuable to assess the mechanisms through which the donor cells recover cognitive function in animal models of CNS disorders. However, it should be reminded that the outcome measurements in animal experiments, including Rotarod and eight-arm radial maze test, may require further development. As recently pointed out by Balkaya et al. [31], the rotarod is a relatively simple and well-evaluated test for short-term (~4 weeks) evaluation of deficits after proximal MCA occlusion in a variety of mouse strains, but may depend on the other factors such as training protocol and motivation. Post-stroke cognitive function may also require several testing to determine the beneficial effects of cell therapy because of its complicated mechanisms.

As aforementioned, the Muse cells have high potential to become to neuronal cells because they differentiate into neuronal cells with very high ratio of ~90% under the presence of cytokine stimulation [14]. They have low telomerase activity and are non-tumorigenic. They comprise 0.03% of bone marrow mononucleated cells in bone marrow aspirate and several percentages of cultured BMSCs [32]. Wakao et al. (2014) have very recently suggested that Muse cells pay an exclusive role in trioblastic differentiation and tissue repair, while non-Muse cells do not directly participate in these events but rather have major roles in trophic and immunosuppressive effects [32]. These findings correlate well with the present histological data. However, the fact that the BMSCs include only several percentages of Muse cells may limit the beneficial effects of BMSC transplantation into the infarct brain. In other words, the therapeutic effects would be enhanced if the Muse cells are isolated or enriched and then transplanted into the infarct brain. Muse cells are quite attractive, because non-tumorigenic stem cells with the ability to generate the multiple cell type of the three germ layers can be obtained through easily accessible BMSCs without introducing exogenous genes [12,13,32]. Further studies are warranted to evaluate whether the therapeutic effect of BMSC transplantation can substantially be improved when Muse and non-Muse cells are combined at a certain best ratio.

## Conclusions

This study demonstrates that among BMSCs, Muse cells and cells other than Muse cells, namely non-Muse cells, may contribute differently to tissue regeneration and functional recovery when they are directly transplanted into the infarct brain. Substantial number of Muse cells remained in the host brain for up to 42 days and expressed neuronal markers Tuj-1 and NeuN, suggesting they replaced the lost neurons, while only negligible number of BMSCs and non-Muse cells remained in the brain at the same time point. Muse and non-Muse groups did not show significant statistical difference in functional recovery, but Muse group was less potential than in BMSC group. Non-Muse cells, however, showed functional recovery at ~2 weeks, and thus they are speculated to exhibit trophic effects to improve the microenvironments of damaged brain at early stage. Since the proportion of Muse cells in BMSCs is only several percentage, tissue repair effect of BMSC transplantation would be enhanced when the ratio of Muse cells are increased, while trophic effect of non-Muse cells might also enhance therapeutic effect synergistically when combined with Muse cells with the best ratio.
